# Emerging Technologies for 6G Communication Networks: Machine Learning Approaches

**DOI:** 10.3390/s23187709

**Published:** 2023-09-06

**Authors:** Annisa Anggun Puspitasari, To Truong An, Mohammed H. Alsharif, Byung Moo Lee

**Affiliations:** 1 Department of Intelligent Mechatronics Engineering and Convergence Engineering for Intelligent Drone, Sejong University, Seoul 05006, Republic of Korea; annisanggun@sju.ac.kr (A.A.P.); totruongan@sju.ac.kr (T.T.A.); 2 Department of Electrical Engineering, College of Electronics and Information Engineering, Sejong University, Seoul 05006, Republic of Korea; malsharif@sejong.ac.kr

**Keywords:** deep learning (DL), emerging technologies, machine learning (ML), reinforcement learning (RL), sixth generation (6G) communication, 6G visions and requirements, wireless communications

## Abstract

The fifth generation achieved tremendous success, which brings high hopes for the next generation, as evidenced by the sixth generation (6G) key performance indicators, which include ultra-reliable low latency communication (URLLC), extremely high data rate, high energy and spectral efficiency, ultra-dense connectivity, integrated sensing and communication, and secure communication. Emerging technologies such as intelligent reflecting surface (IRS), unmanned aerial vehicles (UAVs), non-orthogonal multiple access (NOMA), and others have the ability to provide communications for massive users, high overhead, and computational complexity. This will address concerns over the outrageous 6G requirements. However, optimizing system functionality with these new technologies was found to be hard for conventional mathematical solutions. Therefore, using the ML algorithm and its derivatives could be the right solution. The present study aims to offer a thorough and organized overview of the various machine learning (ML), deep learning (DL), and reinforcement learning (RL) algorithms concerning the emerging 6G technologies. This study is motivated by the fact that there is a lack of research on the significance of these algorithms in this specific context. This study examines the potential of ML algorithms and their derivatives in optimizing emerging technologies to align with the visions and requirements of the 6G network. It is crucial in ushering in a new era of communication marked by substantial advancements and requires grand improvement. This study highlights potential challenges for wireless communications in 6G networks and suggests insights into possible ML algorithms and their derivatives as possible solutions. Finally, the survey concludes that integrating Ml algorithms and emerging technologies will play a vital role in developing 6G networks.

## 1. Introduction

Due to the success achieved by the fifth generation (5G) networks regarding fast and good quality signal transmission compared to the previous generation, the world is currently expecting much faster and better communication in the sixth generation (6G) as the following generation network. However, these attractive and high-demand applications impose challenging key performance indicators (KPIs) and constraints on communication networks, which were also addressed in the International Telecommunication Union Radiocommunication Sector (ITU-R) workshop on International Mobile Telecommunications (IMT) in terms of several usage scenarios and key capability indicators for the 2030 communication network and beyond. These indicators include ultra-reliable low latency communication (URLLC), extremely high data rate, substantially high energy and spectral efficiency, ultra-dense connectivity, secure communication, and massive machine-type communication [1,2,3,4,5]. One of the potential solutions to meet all the requirements is developing emerging technology-assisted wireless communications, which researchers have proposed for the past few years [6,7,8].

**Figure 1:**
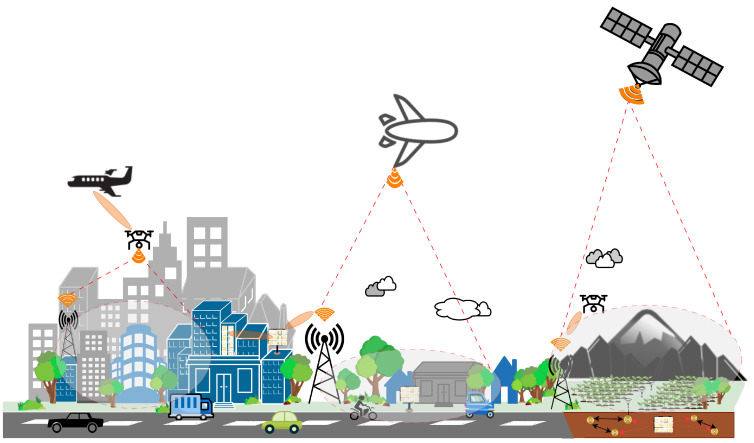
Application of emerging technologies for wireless communication.

Emerging technologies have significantly improved the quality of wireless communications. As shown in Figure 1, emerging technologies can help wireless communication to forward and improve the transmitted signal. Implementation of emerging technologies can provide communication in non-line-of-sight (NLOS) areas [9], dead zone areas [10], disaster environments [11], and even underground and underwater communications [12]. Several researchers have developed studies such as implementing emerging technology to serve NLOS areas and increase throughput [13], unmanned vehicles for information/power transfer [14], secure communication [15], non-orthogonal multiple access (NOMA) applications for interference cancellation [16], and other applications. However, there are some challenges in implementing emerging technology-assisted wireless communication, such as increasing reception of signal diversity from different hardware devices and increasing coexistence requirements, thus giving inaccurate results for model-based approaches. In addition, due to the implementation of massive MIMO (m-MIMO) systems, high overhead and computational complexity becomes a drawback for mathematical models in optimizing the functionality of the physical layer [17]. Therefore, it is likely that the performance enhancement of future wireless networks is difficult to achieve with conventional mathematical solutions.

The application of machine learning (ML) has been gaining traction across a range of industries, including robotics, image processing, healthcare, finance, and transportation [18,19,20,21,22]. In [18], a hybrid of deterministic and swarm-based algorithms was applied for multi-robot exploration in a cluttered environment. In [19], a self-organized and self-healing peer-to-peer information system was designed for a dynamic environment. ML can also be applied for practical applications like finance and healthcare. The use of ML and deep learning (DL) techniques in monitoring and making informed decisions regarding the COVID-19 pandemic was discussed in [20]. Meanwhile, [21] conducted a thorough analysis of COVID-19-related news to predict the stock market using ML technology. Additionally, ML has been employed to predict traffic accident severity, aiming to reduce road accidents and make transportation safer [22].

Other than that, ML and DL have also shown their contribution to optimizing wireless communication. In addition to its ability to work without human intervention, the DL approach is capable of tackling intricate system problems through precise mathematical models [23]. This advanced problem-solving method relies on cutting-edge algorithms that can analyze vast amounts of data, identify patterns, and make accurate predictions. By leveraging DL, the system can achieve more efficient and effective operations, learning to achieve better outcomes. Various ML algorithms and their derivatives have been adapted to improve wireless communication performance [24,25,26,27]. Previous studies of ML demonstrated significant advancements when it was implemented in several emerging technology-aided communications, such as intelligent reflecting surfaces (IRSs), unmanned aerial vehicles (UAVs), autonomous underwater vehicles (AUVs), NOMA, and others.

### 1.1. Related Work

Due to the promising benefits of using ML for future communications, recently, there have been several studies dealing with such implementations [28,29,30,31,32]. In [28], the authors discussed applying different ML types at each communication layer between devices. They highlighted that the ML algorithm used in applications and infrastructure layers is able to meet the 6G requirements, while Tang et al., in [29], have specifically discussed one of the 6G network requirements, URLLC. The authors presented ML abilities for optimizing channel allocation, network routing, congestion control, and adaptive streaming control. In addition, several studies discussed the role of ML algorithms for parameter optimization in m-MIMO communication. In [30], the authors analyzed ML-aided m-MIMO communications for the 5G network. They carried out several issues, including channel estimation, beamforming and precoding, signal detection, distributed and cell-free configurations, and m-MIMO with NOMA. By raising the same communication problem for the 6G network instead of the 5G network, another study focused on the impact of DL algorithm implementation called a transformer, a sequence-to-sequence DL model consisting of encoder–decoder modules and layers for semantic communication [31]. ML algorithms could also be applied to optimize integrated sensing and communication. Demirhan and Alkhateeb, in [32], described ten key roles of ML for integrated sensing and communication, which were divided into three categories: joint sensing and communication, sensing-aided communication, and communication-aided sensing. While the authors of [33] specifically described the reconfigurable intelligent surface (RIS)-aided wireless communication quality improvement due to the implementation of an ML algorithm, reinforcement learning (RL) to be precise, to optimize its communication parameters. They highlighted that implementing RIS as an emerging technology assisted by algorithms based on data statistics could improve communication performance.

Several aforementioned studies have explained some of the ML algorithm capabilities for 6G networks. Yet, there are still very limited studies that specifically discuss the implementation of ML in various emerging technologies based on the 6G requirements approach. The summary of the existing studies for ML implementation in 6G communication networks is shown in Table 1.

### 1.2. Scope and Contributions

Due to the rapid changes and developments in the current environment, ML algorithm implementations allow systems to work adaptively and efficiently. In addition, even though the development of 6G is still in its early stages, it has the potential to revolutionize the way of communication. Emerging technologies such as terahertz (THz) communication, m-MIMO, autonomous vehicles, and optical communication play a critical role in bringing about that communications revolution.

In contrast to recent surveys of ML algorithms implementation for 6G networks, our study delves into the role of ML algorithms in enhancing the efficiency of emerging technologies to meet the demanding demands of the 6G network. Therefore, due to the lack of surveys that focus on the application of ML algorithms in emerging technologies, this research bridges the gap in the current literature by explaining the technical intricacies of the optimization process and highlighting the benefits that can be achieved through the proper implementation of ML algorithms in emerging technologies to overcome various issues in wireless communication and meet 6G network requirements. The list below outlines the main contributions of our research:

- In the beginning, we provide comprehensive details of the visions and requirements for 6G networks. We point out several critical requirements for 6G networks, including zero-energy Internet of Things (IoT), high-speed connectivity and throughput, URLLC, high reliability and availability, security, seamless integration, scalability, and personalizing quality of experience (QoE).
- We provide an insight into the ML algorithm. It includes a brief explanation of the ML algorithms with mathematical approaches. We categorize the different ML algorithms as supervised and unsupervised learning, DL, and RL.
- We extensively analyze the role of ML-aided emerging technologies in empowering this integration by optimizing several parameters in several scenarios of emerging technologies applications, such as IRS, UAV, AUV, NOMA, millimeter-wave (mmWave) and THz communications, free space optics (FSO), visible light communication (VLC), and mobile edge computing (MEC).
- We offer a comprehensive review of the implementation of ML-aided emerging technologies to meet the requirements of 6G communication networks. This study includes several challenges found in 6G KPIs, such as throughput improvement, coverage extension, high reliability, low latency communication, energy efficiency, interconnection of terrestrial and non-terrestrial technologies, sensing and communication, and secure communication.
- In the end, we provide conclusions regarding the impact of ML algorithm implementation on emerging technologies in meeting 6G network requirements.

### 1.3. Organization of the Paper

**Figure 2:**
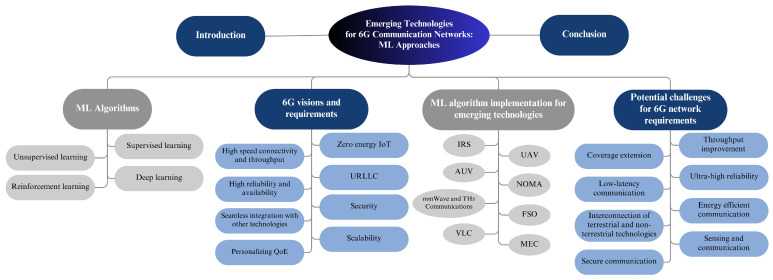
Organization of the paper.

In this paper, we cover various aspects of 6G networks, especially those related to implementing emerging technologies. As shown in Figure 2, the rest of this paper is structured as follows. A comprehensive discussion of the visions and requirements for 6G networks is outlined in Section 2. Then, we provide an overview of the ML algorithms in Section 3. Additionally, we furnish comprehensive details on the ML algorithms deployed for emerging technologies in Section 4. Furthermore, we examine the potential challenges that future wireless communication may encounter concerning the requirements for 6G networks, as well as several insights for future research opportunities in Section 5. Finally, Section 6 presents the conclusions of the paper.

## 2. 6G Visions and Requirements

In this section, an overview of the key elements and features expected in 6G networks will be provided. The success of 5G in enhancing communication has raised high expectations for 6G. Additionally, the anticipated involvement of a massive number of users and connectivity in the 6G network further contributes to these expectations. It was shown by the KPIs of 6G networks that have already been announced, which was also discussed at the ITU-R workshop on IMT. Several requirements for 6G networks need to be considered, including zero-energy IoT, high-speed connectivity and throughput, URLLC, high reliability and availability, security, seamless integration, scalability, and personalizing QoE.

### 2.1. Zero-Energy IoT

Zero-energy IoT is a new technology that allows IoT devices to operate without batteries. Instead, the energy necessary for communication is harvested from the surroundings. This could be performed through various means, such as the energy from vibrations, sunlight, temperature gradients, and radio waves that can be converted into electricity. Implementing 6G networks using zero-energy IoT could give several benefits, such as environmental impact reductions and lower costs. Zero-energy IoT devices do not require batteries, making them significantly cheaper, and there will be no waste when they are disposed of [34]. Furthermore, the use of zero-energy IoT will reduce the possibility of failure due to its characteristics, which are not reliant on batteries. Other than that, 6G networks are expected to be more energy efficient than 5G networks, making them more suitable for zero-energy IoT devices.

Zero-energy IoT has the potential to revolutionize the IoT industry by making it possible to deploy large numbers of low-cost, low-power devices that can be used to collect data in a variety of environments [35,36]. This could lead to new applications in areas, such as smart cities, Industry 4.0, and agriculture.

### 2.2. High-Speed Connectivity and Throughput

High-speed connectivity and throughput are two key features of 6G networks. 6G is expected to offer peak data rates of up to 1 Tbps, 1000 times faster than 5G. There are a number of technologies that are being considered for use in 6G networks to achieve high-speed connectivity, including THz frequencies [37]. THz frequencies offer a much wider bandwidth than those used by 5G networks, enabling peak data rates of up to 1 Tbps. m-MIMO and beamforming could also be implemented to improve wireless channel efficiency, SNR, and data rates. Other than that, a full duplex is another potential technology that can double the data rate of the wireless channel by allowing a device to transmit and receive data simultaneously.

Therefore, implementing high-speed connectivity could give some benefits to the 6G network, such as enabling a more immersive and interactive user experience [38]. High-speed connectivity technologies could also help to improve efficiency by allowing them to transfer data more quickly and easily [39]. Furthermore, it can help network security improvement by making it more difficult for attackers to exploit vulnerabilities [40,41].

### 2.3. URLLC

URLLC is a type of communication that is characterized by its high reliability and low latency. This means that URLLC is well-suited for applications that require real-time communication and where even a small amount of data loss or delay can be critical. As the development of 6G continues, more innovative technologies being used to achieve the high reliability and low latency requirements of URLLC are expected. It is supported by the advantages of URLLC for 6G networks. A URLLC could improve the safety of critical infrastructure and systems by ensuring that they are able to communicate reliably and with low latency [42]. It will also increase efficiency by enabling users to automate processes and make better decisions in real-time.

Other than that, because it prioritizes reliability and latency, URLLC could use less power and bandwidth than other communication technologies [43]. This means that URLLC networks are optimized to deliver small amounts of data quickly and reliably, even in challenging conditions [44]. Those benefits of URLLC make it suitable for several advanced technologies, such as AI-driven optimization, which will optimize the URLLC networks in case of predictive analytics, resource allocation, security, and network troubleshooting.

### 2.4. High Reliability and Availability

High reliability and availability are also critical requirements for 6G networks. High availability refers to the ability of a network to remain operational even in the event of failure. This is essential for 6G networks, as they will be used to support a wide range of critical applications. There are several factors that can contribute to high reliability and availability in 6G networks, including redundancy, load balancing, failover, and monitoring. Redundancy is the use of multiple components to perform the same function, while failover is the ability of a network to switch to a backup component automatically. That approach guarantees that if one component malfunctions, another component can seamlessly assume control and sustain the intended functionality [45]. Moreover, load balancing and monitoring help prevent any component from becoming overloaded or causing a failure by distributing traffic across multiple components and monitoring the network health tracking process [46].

By implementing those and other measures, 6G networks can be made highly reliable and available [47]. It will ensure that they can continue to provide critical services even in the event of failure [48]. Therefore, it could lead to reducing the likelihood of service outages, increasing the uptime of 6G networks, providing longer periods of time, enhancing security, and improving user experience by ensuring that users are able to access services even if there is a failure [49,50].

### 2.5. Security

Security communication in 6G networks is a critical issue, as the network will be used to transmit sensitive data. Secure communication could help improve the 6G network’s security, privacy, trust, and user experience by preventing users’ data from being accessed by unauthorized parties [51,52]. It will challenge the attackers to eavesdrop on or intercept the communication, ensuring that communications between users and service providers are secure and confidential, and reducing the risk of security incidents.

However, there are some security challenges that need to be addressed in 6G networks. 6G networks will have a larger attack surface than the previous generation of networks because they will use a wider range of frequencies and technologies [53,54]. However, in contrast, it will make the 6G network more complex than previous network generations, which makes it more difficult to secure them, as there will be more potential vulnerabilities to exploit [55]. As 6G networks become more sophisticated, new attack vectors will emerge. These could include attacks on the network infrastructure, the devices that connect to the network, or the data that is transmitted over the network.

### 2.6. Seamless Integration

Due to the need for 6G networks to seamlessly integrate with existing 5G networks, as well as other networks such as Wi-Fi and Ethernet, the seamless integration of 6G networks is an essential requirement for 6G network success. This will allow users to seamlessly switch between networks as they move around, and will also allow for the efficient use of spectrum. Furthermore, the interconnection of terrestrial and non-terrestrial technologies that are expected to be implemented in 6G networks increasingly makes seamless integration even more necessary.

Terrestrial and non-terrestrial technologies are both being considered for use in 6G networks. Terrestrial technologies, such as mmWave, m-MIMO, and beamforming, use the Earth’s surface to transmit and receive signals. In contrast, non-terrestrial technologies, such as satellites, use the atmosphere or space to do so [56]. Those technologies will allow 6G networks to provide global coverage, high data rates, and low latency [57]. Terrestrial technologies can provide coverage in urban areas, while non-terrestrial technologies can provide in rural areas and remote locations [58,59,60]. Non-terrestrial technologies could also provide high data rates and low latency, which is essential for applications such as virtual reality (VR) and augmented reality (AR) [61]. Other than that, by interconnecting terrestrial and non-terrestrial networks, 6G networks can be made more secure [62]. This is because non-terrestrial networks are less susceptible to physical attacks than terrestrial networks. However, the interconnection of terrestrial and non-terrestrial technologies in 6G networks is a complex challenge due to issues such as heterogeneity, mobility, and security.

### 2.7. Scalability

6G networks are expected to have better scalability for machine-to-machine (M2M) connections than the previous networks. Scalability is the ability of a network to handle an increasing number of users and devices without sacrificing performance. The 6G networks will characterized by higher data rates, lower latency, and massive connectivity. These features will make them well-suited for M2M applications. M2M refers to the communication between devices without human intervention. In order to support the growing number of M2M connections in 6G networks, scalability should be one of the things to be considered.

As the number of M2M connections increases, the need for scalable networks will also increase. Thus, 6G networks are well-positioned to meet this need due to massive devices and connectivity adoption that will allow for a much larger number of M2M devices to be connected to the network, which will be essential for applications such as IoT. Scalability will also help to improve the user experience by ensuring that users have a reliable and consistent connection to the network. This could enable new and innovative M2M applications in wireless networks, such as smart cities, industrial automation, and transportation.

There are a number of factors that contribute to scalability in 6G networks, including heterogeneous networks, network slicing, software-defined networking (SDN), hybrid cloud, and AI [63,64,65]. Heterogeneous networks and network slicing could help to scale the network as needed to support massive applications and services [66]. SDN will allow for the network to be controlled and managed by the software, which makes the network easier to adapt to changes in traffic demand [67,68]. A hybrid cloud is a deployment model that combines the benefits of the public cloud and private cloud. The public cloud can scale the network horizontally by adding more resources, while the private cloud can scale the network vertically by adding more powerful resources [69,70]. Thus, combining them could be useful for applications that experience sudden spikes in traffic and require a lot of processing power [71]. A hybrid cloud allows organizations to have the flexibility to choose the right cloud environment and scale the network as needed, in order to provide high performance for demanding applications [72]. Additionally, AI can be used to optimize the performance of the network and to identify and mitigate potential problems. Therefore, scalability is essential to support the growing demand for connectivity and the increasing number of devices that will be connected to the 6G networks.

### 2.8. Personalizing QoE

The QoE in 6G wireless networks is expected to be significantly improved over previous generations. This is due to a number of factors mentioned in the subsection above, such as higher data rates, lower latency, wider coverage, more reliability, and better security [73]. However, in order to enhance the overall efficiency of the network towards a specific objective, it is imperative that QoE be personalized for each user or application on the network, so that the network operators can ensure that resources are used efficiently. Besides that, 6G networks are expected to support a wide range of users and applications with different requirements for QoE. Thus, personalizing QoE can ensure that users are able to optimize their experience and achieve the best possible experience in a timely and effective manner [74]. Therefore, prioritizing the personalization of QoE is a critical component of achieving success in the upcoming 6G network.

There are several promising technologies that can be implemented in order to improve QoE, including network slicing, edge computing, ML, and AI technologies. AI and ML can personalize network experiences by providing and analyzing specific data for user behaviors and preferences [75,76]. Network slicing could help to personalize QoE by dividing the network into multiple virtual networks, where each virtual network can be customized to meet the specific needs of the users or applications [77]. In addition, edge computing can be used to bring computing resources closer to the end users, which leads to a better QoE [78,79]. Implementing radio access technologies, such as THz communication, could also support more demanding applications and provide a better QoE for users, such as higher data rates or lower latency [80,81]. Personalizing QoE would be very useful to be implemented in specific cases, such as VR, AR, self-driving cars, Industrial IoT, and smart cities.

**Figure 3:**
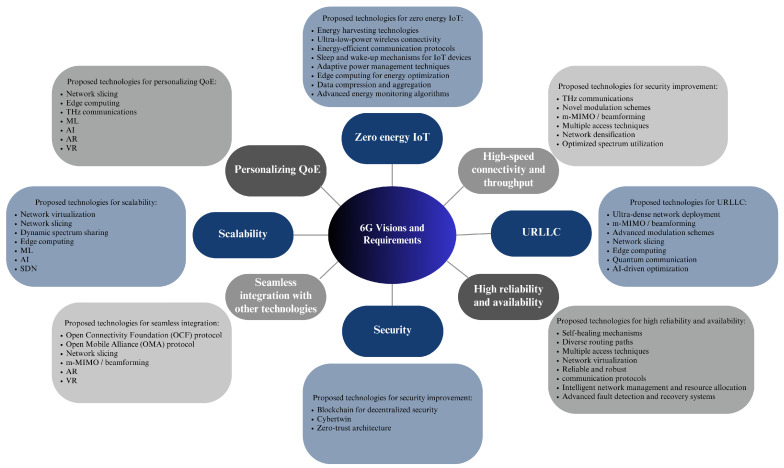
6G visions and requirements.

Figure 3 shows 6G visions and requirements, including its potential technologies as discussed in this subsection.

## 3. ML Algorithms

ML is a branch of artificial intelligence that uses mathematical algorithms to discern trends and patterns within complex, multi-dimensional datasets. A key component of ML’s effectiveness is its ability to learn from the data itself, which allows it to automatically adapt over time and improve its performance. Due to its versatility, effectiveness, and ability to address complex problems without requiring explicit programming instructions, ML has been incorporated into a variety of applications, including image and speech recognition, medical diagnosis, recommendation systems, financial forecasting, and many others. Therefore, ML has emerged as a transformative technology, revolutionizing industries and paving the way for numerous advancements and innovations in modern society.

There are several techniques within the ML domain, including supervised learning, unsupervised learning, DL, and RL. A supervised learning approach employs labeled data to make accurate predictions. Conversely, unsupervised learning algorithms can uncover patterns in unlabeled data. By utilizing neural networks (NN), DL methods extract hierarchical representations from the data. In contrast, RL trains models to make sequential decisions through interactions with the environment. These diverse approaches collectively provide a comprehensive toolkit for addressing a wide range of challenges in both the research and practical applications of ML.

### 3.1. Supervised Learning

In ML, supervised learning involves mapping input data to output data with high accuracy. This approach requires labeled datasets to train the model, and is commonly used for classification and regression problems. Some of the techniques used in supervised learning include linear and logistic regressions, decision trees, random forests, gradient boosting, and support vector machines (SVM).

#### 3.1.1. Linear Regression

Linear regression is one of the most popular ML algorithms, due to its ability to predict continuous variables easily. Linear regression works based on the relationship between the target variable (dependent variable) and the predictor variable (independent variable). A sloped straight line of regression shows the relationship between these variables. It can be a negative linear relationship (the dependent variable decreases while the independent increases) or a positive one (both variables increase). Thus, the mathematical representation is written in Equation (1) [82].

(1) y=bx+c

where *y* represents the dependent variable, *x* represents the independent variable, *b* represents the slope of the line, and *c* represents the intercept of the line. Meanwhile, to determine the accuracy of the predicted value, linear regression uses the mean squared error (MSE) cost function, written in Equation (2).

(2) $$MSE=\frac{1}{N}\sum_{i=1}^{n}{\left(y_{i}-\left(bx+c\right)\right)}^{2}$$

where *N* represents the total number of observations.

#### 3.1.2. Logistic Regression

The logistic regression model is widely used to predict binary outcomes based on probabilities. In contrast to linear regression, which assumes a linear relationship between predictors and the target variables, logistic regression uses a sigmoid or S-shaped logistic function to reflect the non-linear relationship between predictors and the likelihood of a specific result. The logistic regression can be given as follows:

(3) $$f\left(x\right)=\frac{1}{1+e^{-x}}$$

where f(x) represents the predicted probability of the binary outcome, *x* denotes the linear combination of predictor variables and their corresponding coefficients, and e is the base of the natural logarithm.

A threshold value is applied to the predicted probabilities in order to classify the binary outcome. Traditionally, the threshold is set at 0.5, with predictions above 0.5 classified as 1 (positive outcome), and predictions below 0.5 classified as 0 (negative outcome). The threshold can, however, be adjusted according to the specific requirements of the problem to achieve the appropriate balance between sensitivity and specificity.

In logistic regression, the predicted outcome is obtained by comparing the predicted probability to the threshold value. For example, if the predicted probability is greater than a threshold, it is classified as 1, and if it is less than a threshold, it is classified as 0 [82].

#### 3.1.3. Decision Tree

Decision trees are a popular supervised ML technique used for both classification and regression problems. They provide an intuitive and interpretable approach by representing data in a tree-like structure. In this structure, the root node represents the entire dataset, the branches correspond to decision rules based on attribute values, and the leaves represent the output or prediction [83].

Attribute selection is a critical step in constructing decision trees. The goal is to determine the most informative attributes that effectively split the data to maximize predictive accuracy. Two commonly used attribute selection measures are the Gini index and information gain.

The Gini index measures the impurity or disorder of a node in a decision tree. It calculates the probability of a specific attribute being incorrectly classified. The Gini index and information gain are mathematically represented in Equations (4) and (5), respectively.

(4) $$Gini=1-\sum_{i=1}^{n}p_{i}^{2}$$

(5) InformationGain=E(S)−[WE(s)]

(6) $$E\left(S\right)=\sum_{i=1}^{n}-p_{i}\log_{2}p_{i}$$

where $p_{i}$ represents the probability that a feature is classified as class *i*, *W* represents the weighted average, and E(S) and E(s) indicate the entropy of the main node and each feature, respectively.

The mathematical formulations of the Gini index and information gain provide a solid foundation for attribute selection in decision trees. These measures allow decision trees to effectively partition the data and make informed decisions at each node, leading to accurate predictions. Decision trees’ interpretability and ability to handle both categorical and numerical data make them valuable tools for various applications in various domains.

#### 3.1.4. Random Forest

Similar to the decision tree technique, the random forest technique is widely used for classification and regression problems in ML. A random forest leverages the concept of ensemble learning by combining multiple decision trees to make predictions. This approach harnesses the collective wisdom of multiple models to enhance the accuracy and robustness of predictions [83].

In a random forest, each decision tree is constructed independently, utilizing a subset of the training data and a random selection of features. This sampling process, known as bootstrap aggregating or “bagging”, introduces diversity among the trees. By aggregating the predictions from all the individual trees, the random forest predicts the final output based on the majority vote (for classification) or the average (for regression) of the predictions generated by the constituent trees.

The random forest algorithm offers several advantages over a single decision tree. Firstly, it reduces the risk of overfitting, as the averaging of multiple models helps to mitigate the effects of noise and biases in the training data. Additionally, by randomly selecting a subset of features for each tree, a random forest introduces feature diversity and reduces the influence of dominant features, leading to a more balanced and robust model.

The number of trees in a random forest is a crucial parameter that impacts the model’s performance. As the number of trees increases, the random forest becomes more capable of capturing complex patterns and relationships in the data. However, there is a trade-off between predictive accuracy and computational efficiency, as the inclusion of more trees typically results in longer computation times.

The random forest technique has been extensively applied in various domains, including finance, healthcare, and image analysis. It has demonstrated its effectiveness in tackling complex problems, such as fraud detection, disease diagnosis, and object recognition.

Ensemble learning is an ML approach that seeks better prediction by combining multiple models. In general, there are four methods of ensemble learning which are bagging, boosting, staking, and a mixture of experts.

- Bagging: a technique that generates multiple training data subsets and trains the model on each subset, then combines the output;
- Boosting: a method that creates multiple models where each model is trained on a modified version of the training dataset;
- Stacking: a method that generates bootstrapped data subsets and adds a meta-classifier at the end of the process to rectify any incorrect behavior from the initial classifiers;
- Mixture of experts: a technique that utilizes a whole dataset for each classifier input. A gating network is applied to produce weights for each initial classifier before going through a linear combination.

#### 3.1.5. Gradient Boosting

Gradient boosting is a supervised ML that takes the concept of boosting method of ensemble learning. This algorithm is designed to solve both classification and regression problems by combining multiple weak learners into strong learners.

In gradient boosting, the algorithm iteratively builds a sequence of weak learners, where each learner is trained to correct the errors of the previous model’s predictions. At each iteration *i*, the algorithm fits a decision tree to the negative gradient of the loss function, aiming to minimize the residuals or errors of the previous model’s predictions. Gradient boosting in mathematical representation is shown in Equation (7).

(7) $$f\left(x\right)=\sum_{i}\alpha_{i}h_{i}\left(x\right)$$

where f(x), $\alpha_{i}$, and $h_{i}$ represent strong learners, the weight of the last iteration, and weak learners, respectively.

Each weak learner is designed to capture a specific aspect or pattern in the data that the previous models may have missed. By iterative training and combining these weak learners, gradient boosting gradually improves its predictive performance, reducing the overall error or loss. The choice of loss function depends on the problem at hand. For example, in classification problems, the cross-entropy loss or exponential loss may be used, while in regression problems, mean squared error or mean absolute error could be employed.

Gradient boosting has gained significant attention and popularity in various domains due to its ability to handle complex problems and deliver high predictive accuracy. It has proven successful in diverse applications such as click-through rate prediction, anomaly detection, and recommendation systems.

#### 3.1.6. Support Vector Machines (SVMs)

SVMs are powerful and versatile ML algorithms that have gained considerable attention in the field of supervised learning. They belong to the class of discriminative classifiers, and are widely used for both classification and regression tasks [83]. SVMs have proven to be effective in various domains, including image recognition, text categorization, and bioinformatics.

The fundamental concept behind SVMs is to find an optimal decision boundary or hyperplane that maximally separates the data points belonging to different classes. The key idea is to identify a decision boundary with the maximum margin, which represents the distance between the boundary and the closest data points of each class. This property makes SVMs robust and less susceptible to overfitting.

SVMs excel in scenarios where the data is not linearly separable in the original feature space. To address this, SVMs employ a technique called the “kernel trick”, which implicitly maps the input data into a higher-dimensional feature space where linear separation becomes feasible [83,84]. This allows SVMs to capture complex, nonlinear relationships between the input features and the target variable.

It is necessary to find the optimal hyperplane when training an SVM in order to maximize the margin and minimize the classification error. It is common for convex optimization techniques to be used in order to solve this optimization problem. An SVM’s generalization performance is influenced by the support vectors, which are the data points closest to the decision boundary.

Moreover, SVMs can handle both binary and multi-class classification problems. Binary classification involves separating data into two classes, while multi-class classification extends the SVM framework to handle multiple classes by using strategies such as one-vs-one or one-vs-rest.

There are several advantages to using SVMs, including their ability to handle high-dimensional data and their robustness against overfitting. As well as providing a clear sense of decision boundary, SVMs can also be used to gain insights into the classification process, because they provide a clear sense of the decision boundary.

### 3.2. Unsupervised Learning

The Unsupervised Learning type of ML is trained using no pre-existing labels and input data that is not classified, in order to discover patterns within the data. Thus, it does not need external supervision to learn the data, and does not have a predefined output.

#### 3.2.1. K-Means Clustering

The K-means algorithm, also known as the K-nearest neighbors algorithm, is a method of clustering data instances based on pairwise distances between them. This algorithm is aimed at minimizing the variance between clusters.

Initially, the algorithm partitions the input points into K initial sets. The sets can be randomly assigned or determined by heuristic methods based on the data. The centroid is the mean or center of its clusters, whose values are updated for each iteration *i*, where the initial centroids of k clusters are chosen randomly. The objective function of K-means clustering *P* is shown as follows:

(8) $$\left(P\right)min\sum_{j=1}^{k}\sum_{i=1}^{n}{∥x_{i}^{j}-c_{j}∥}^{2}$$

where $∥x_{i}^{j}-c_{j}∥$ represents the distance function.

The number of clusters is a critical parameter in K-means clustering. A large number of clusters may improve data separation, but it can also lead to overfitting. The Elbow method is a popular technique for determining the optimal number of clusters in K-means clustering. The within-cluster sum of squares (WCSS) plotting can be used to determine the optimal number of clusters, where the optimal number of clusters is the point at which the WCSS decreases sharply.

#### 3.2.2. Hierarchical Clustering

Hierarchical clustering differs from K-means in that it allows the number of clusters to change during each iteration. It can be divided into two categories: divisive clustering and agglomerative clustering. The divisional clustering algorithm starts with all data instances grouped into a single cluster, and then splits them in each iteration, resulting in a hierarchical cluster structure. Agglomerative clustering, on the other hand, requires a bottom-up approach, where each instance is considered a separate cluster and is merged iteratively. Regardless of the method used, the resulting hierarchy will have N levels, where N represents the total number of instances.

Hierarchical clustering, in contrast to other clustering methods, does not provide a single definitive clustering solution for the data. Instead, it generates N−1 clusterings, leaving it up to the user to determine the most suitable one for their specific objectives. To aid in this decision-making process, statistical heuristics are sometimes employed.

After the training phase, the resulting arrangement of clusters forms a hierarchical structure, often visualized using a dendrogram. In the dendrogram, nodes represent clusters, and the length of an edge connecting a cluster to its split reflects the dissimilarity between the resulting split clusters. Dendrograms have contributed to the popularity of hierarchical clustering, as they offer an easily interpretable visualization of the clustering structure.

It should be noted that selecting the appropriate clustering solution from the hierarchical structure requires careful consideration, and may involve domain knowledge and expertise. The dendrograms serve as a valuable tool in understanding and interpreting the clustering outcomes.

The use of hierarchical clustering and the interpretation of dendrograms have found wide applications across various domains due to their ability to provide an intuitive and accessible view of the clustering structure

#### 3.2.3. Principal Component Analysis (PCA)

PCA is a popular unsupervised ML technique widely used for dimensionality reduction and data analysis. It aims to transform high-dimensional data into a lower-dimensional space while retaining maximum information.

The key objective of PCA is to identify the underlying structure or patterns within the data. It achieves this by splitting the data into a k-dimensional space based on the principal components, which are the eigenvectors of the covariance matrix. Each principal component captures a different aspect of the data’s variability.

The eigenvalues associated with the principal components represent the variances explained by each component. Higher eigenvalues indicate a greater proportion of the total variance explained by the corresponding principal component.

By leveraging PCA, analysts and researchers can gain insights into the essential features and relationships within complex datasets while reducing the dimensionality. This technique has found widespread application across various fields, including image processing, genetics, and finance, among others.

### 3.3. Deep Learning (DL)

DL is a sub-branch of ML consisting of multiple NN layers that can be implemented for data prediction, classification, or other data decision-making by learning its representations. The structure of DL consists of input, output, and hidden layers. Based on the forward-propagation cycle, the neurons in every hidden layer calculate the weighted sum of the input of the previous layer and forward it to the following layers using a nonlinear activation function. DL converts the raw data into pairs of nonlinear input–output mapping used for executing actions to achieve the objective. While learning the characteristics of the raw data in high complexity, each layer of NN will transform to a higher level.

#### 3.3.1. Artificial Neural Networks (ANNs)

ANNs are fundamental neural network models, often referred to as feed-forward neural networks. They comprise a group of interconnected neurons organized in layers, where information propagates in a unidirectional manner, from the input layer through intermediate hidden layers (if present) to the output layer [85].

In an ANN, the input data is processed only in the forward direction, with each neuron receiving input from the previous layer and generating an output that becomes the input for the subsequent layer. The hidden layers, which may or may not be included in an ANN model, provide a means for the network to learn and capture complex representations of the input data [83].

The absence of hidden layers in an ANN simplifies its operation and interpretation. Without hidden layers, ANNs primarily function as linear models, with input data being mapped directly to the output layer. This characteristic makes ANNs particularly suitable for problems that involve linear relationships and straightforward decision-making processes.

The simplicity of ANNs, both in terms of their structure and interpretability, has contributed to their widespread usage and understanding in various domains. Researchers and practitioners often employ ANNs as a starting point to explore more complex neural network architectures and advanced ML techniques.

#### 3.3.2. Deep Neural Networks (DNNs)

DNNs represent the most widely implemented algorithm in DL. DNNs are characterized by their fully connected structure, where multiple layers are stacked, and each neuron is connected to all neurons in the preceding and following layers. The architecture of a DNN allows for the extraction of increasingly complex representations as information flows through each layer. This hierarchical representation learning enables DNNs to capture intricate patterns and relationships in the data [86].

Optimizing the learning performance of DNNs is crucial, and a key consideration is the weight of the model. The weights determine the strength of connections between neurons, and play a vital role in the network’s ability to accurately learn from the data. Careful adjustment of the weights is necessary to prevent issues such as vanishing or exploding gradients, which can hinder the training process [85].

Efficient weight initialization, regularization techniques, and appropriate optimization algorithms are employed to ensure effective weight management in DNNs. These practices contribute to enhancing the learning capacity and overall performance of DNN models.

Due to their ability to handle complex data and learn intricate representations, DNNs have achieved remarkable success in various domains, including computer vision, natural language processing (NLP), and speech recognition. Their flexibility and versatility have made DNNs a powerful tool for solving challenging problems and advancing the field of DL.

#### 3.3.3. Convolutional Neural Networks (CNNs)

CNN architecture focuses on identifying similarities within 2D feature vectors. Typically, CNN models start with convolutional layers, followed by nonlinear activation functions, pooling layers, and additional convolutional layers. The fundamental concept underlying CNN architecture is local connection and weight sharing.

Unlike DNNs that employ a fully connected structure, a CNN’s convolutional layers have each unit connected to a local patch in the preceding layer, and all connections within the patch share the same weight matrix. This weight-sharing property significantly reduces the number of learnable parameters in CNNs [83].

By leveraging local connections and weight sharing, CNNs excel in capturing local patterns and spatial relationships in data. This makes them highly effective in tasks such as image recognition and computer vision, where identifying local features is crucial.

The architecture of CNNs enables them to automatically learn hierarchical representations from raw data, starting from low-level features and progressively extracting more complex and abstract features. This hierarchical feature learning contributes to CNNs’ ability to achieve impressive performance in various domains.

#### 3.3.4. Recurrent Neural Networks (RNNs)

RNNs differ from DNNs and CNNs in that they process input sequences iteratively and have the ability to retain information from the past, thus avoiding memory loss. The architecture of an RNN can vary depending on the specific application it is designed for.

In an RNN, the hidden layers share parameters across different time steps, similar to the weight-sharing technique used in CNNs. This shared structure reduces the model’s complexity and helps prevent overfitting. However, training RNNs using backpropagation through time can present challenges. The backpropagation algorithm, which leverages a stochastic gradient descent, unfolds in time and can impede the smooth flow of information, leading to difficulties in training.

To address the issues of vanishing or exploding gradients, and to enhance the memory capabilities of traditional RNNs, Long Short-Term Memory (LSTM) networks were introduced as a robust alternative. LSTM networks incorporate specialized memory cells that enable them to selectively retain and forget information, making them more effective in capturing long-range dependencies in sequential data. LSTM networks have gained significant popularity due to their ability to overcome the limitations of traditional RNNs and provide improved memory and learning capabilities.

The utilization of RNNs and LSTM networks has led to significant advancements in various domains, including NLP, speech recognition, and time series analysis. Their ability to model sequential data and capture temporal dependencies makes them well-suited for tasks involving dynamic patterns and contextual information.

### 3.4. Reinforcement Learning (RL)

In recent years, ML has played an important role by allowing machines to make decisions automatically based on their datasets. RL is an advancement in ML, specifically DL. As derivatives of ML, RL algorithms allow machines to interact with a dynamic environment while considering their experience dataset to make the most accurate decision [87,88,89]. Based on the Markov decision process (MDP) formula, the RL algorithm consists of three stages: state, action, and reward.

- State: A set of environment’s characteristics (*S*) received by the agent provided by the environment. $s_{1}$ represents the initial state and the environment for each time step *t* indicated by $s_{t}\in S$;
- Action: a set of actions taken by the agent (*A*) in response to the characteristics of the environment, while next state $s_{t+1}$ indicates the latest environmental characteristics sent to the agent each time the agent executes an action $a_{t}\in A$ in a time instant *t*;
- Reward: A set of feedback provided by the environment based on the action given by the agent. When the result obtained are better than those previously achieved, the environment will give a reward $r_{t}$ to the agent for every time instant *t*. In contrast, a punishment will be given when the results obtained are worse than the previous ones;
- Q-Value function: a state–action value function Q(s,a) received by the agent that indicates the level of action $a_{t}$ we took for each given state $s_{t}$.

RL methodology can be classified as policy-based or value-based, based on the approach taken to decide what action to take [90]. The value-based method considers the optimal Q-value $Q^{*}\left(s,a\right)$, while the policy-based considers the optimal policy value or transition probability $\pi^{*}\left(s,a\right)$.

The combination of DNN and RLs is beneficial in solving intricate problems. The DNN could act as a Q-value estimator Q(s,a) in the value-based Deep RL (DRL), as in Equation (5). In addition, it could also perform as a gradient estimator ${▿}_{\theta }J\left(\theta \right)$ to estimate the probability value of J(θ) in the policy-based method, as shown in Equation (6).

(9) Q(s,a;θ)≈Q(s,a)

(10) $${▿}_{\theta }J\left(\theta \right)\approx \sum_{t\geq 0}r\left(\tau \right){▿}_{\theta }\log\pi_{\theta }\left(a_{t},st\right)$$

where θ represents the weight of the DNN, r(τ) indicates the reward for each trajectory (path), and $\log\pi_{\theta }\left(a_{t},st\right)$ indicates the probability of the performed action in each state. Linear in its development, a study has recently been carried out on the application of DRLs in various branches of technology, one of which is emerging technology.

Due to its characteristic that works based on each environment, RL is useful in a constantly changing environment and suitable to handle very large and complex data at the cost of the computation. In contrast, it will be unavailing for simple problems because it will be hard to achieve the maximum reward. Furthermore, RL is highly dependent on their reward function quality, and it is difficult to debug and interpret RL implementation.

Based on the discussion in this section, Table 2 shows the comparison of each ML algorithm in terms of their concept, advantages, and limitations for their implementation.

## 4. ML Algorithm Implementation for Emerging Technologies in a 6G Network

### 4.1. Intelligent Reflecting Surfaces (IRSs)

IRS or RIS is a technology that has been intensively discussed by researchers to support 6G communication networks because of its ability to improve signal quality by working passively and having low installation and maintenance costs. The IRS is an artificial two-dimensional planar metasurface that has reconfigurable features implemented through electronic circuits. IRS helps transmit data and avoid NLOS propagation in wireless communications by reflecting electromagnetic waves (EMs) to the desired receiver to enhance the transmission quality of service (QoS) significantly. Several things in IRS-aided communication need to be considered to support the QoS obtained, such as channel state information (CSI), phase shift configuration, beamforming, power and spectral efficiency, and physical layer security. These issues can be overcome by optimizing using ML. Table 3 provides a concise summary of the studies that are discussed in this subsection.

The study in [91] applied two DNNs to find the relationship between the pilot signals, the optimal phase shift, and the downlink transmit beamforming vector. The proposed system was shown to reduce pilot overhead while still providing performance comparable to communication with perfect CSI. Whereas in [92], the optimum IRS phase shift and overhead reduction are obtained by implementing the CNN architecture. The system can converge to near-optimal data rates using less than 2% of the receiver locations. Applications of ML to maximize spectral efficiency have been applied in [93,94,95]. The system proposed by authors of [93] achieved almost the same performance as the alternative optimization method with less computational complexity by using a learning phase-shift NN (LSPNet) that is trained using an unsupervised learning method. Other than that, in [94,95], the system improved spectral and power efficiencies by applying a DL-based framework in RIS-assisted MIMO and MIMO–NOMA communication systems with STAR-RIS, respectively. The approach suggested in [94] can configure real-time phase shifts, improve rate performance in low signal-to-noise ratios (SNRs), and provide higher energy efficiency (EE) than the optimal beamforming solution. In comparison, the DL-based framework in [95] provided the low complexity iterative algorithm with guaranteed convergence at a relatively optimal level, and predicted the optimal user’s power allocation and phase shift configuration at STAR-RIS.

Another study focused on minimizing transmit power in an RIS-assisted MISO-OFDM system by implementing a DRL-based framework, which is a twin delay deep deterministic policy gradient (TD3) algorithm [96]. The system was shown to be effective in reducing transmit power, which is almost the same as the lower bound obtained by the manifold optimization algorithm, but with a much shorter computation time. Another crucial issue for 6G communication networks is privacy and security. Several works have explored the application of ML in IRS-aided PLS communications. In [97], the authors aimed to enhance the efficiency and the learning convergence rate by implementing post-decision state (PDS) and prioritized experience replay (PER) schemes. The result outperformed the deep q-learning (DQN) method by increasing the system’s secrecy rate as well as the probability of satisfied QoS, while the authors of [98] were optimizing the average secrecy rate and throughput in IRS-assisted secure buffer-aided cooperative networks. The proposed multi-agent DRL (MA-DRL) method significantly improved those two parameters over the max-ratio algorithm.

### 4.2. Unmanned Aerial Vehicles (UAVs)

UAVs are one of the most widely applied unmanned vehicles, and have the potential for future communications. UAV-aided communications have become increasingly popular for communications applications in recent years due to several advantages they offer, such as mobility, high maneuverability, low-cost maintenance, and easy deployment [99]. Their ability to hover and move around an area allows communication to occur in an infrastructure lacking due to NLOS. By optimizing various parameters, such as the UAV trajectory, UAV placement, bandwidth, and power allocation, the performance of a UAV-assisted communication network can be significantly improved. This can lead to a number of benefits, such as increased coverage, improved QoS, and reduced costs.

In [100], the authors developed an RL approach to allow a UAV to traverse a given trajectory autonomously. The proposed system gave fewer localization errors compared to other methods mentioned in the study by considering the fixed amount of UAV energy consumption, path length, flying time, and velocity, while in [101], the authors have implemented a MA q-learning algorithm, ESN algorithm, for placement optimization, trajectory acquisition, and power control. The proposed ESN algorithm predicted the user’s movement at high accuracy and provided a high quality of maintaining the trajectory and power control. Another study focused on UAV path planning and obstacle avoidance by implementing a DQN-based algorithm [102]. The proposed modified q-learning showed reducing 50% in computation time and 30% of the path length than the state–action–reward–state–action (SARSA) algorithm. Another algorithm, called DL-based energy optimization (DEO), has been proposed to optimize energy for edge devices in [103]. It is used to dynamically adjust the emission energy of the edge device so that the received power of the UAV is equal to the receiver’s sensitivity. They used DL to predict the UAV location information. The results showed that the DEO algorithm achieved a weighted mean absolute percentage error (WMAPE) of less than 2% under the effect of a communication delay of less than 1 s.

In [104], the authors were concerned about the required energy in a moving UAV. They used the mean-fielded game (MMFG) method to obtain the optimal trajectory and proposed the mean-field trust region policy optimization (MFTRPO) algorithm, which proved to be effective in robustness and superiority in energy efficiency. Furthermore, ML can be used for UAV-aided communication for resource allocation and handover management. The authors of [105] presented an algorithm for handovers and radio resources management (H-RRM) in UAV communications. They used DQN to make decisions about the way to allocate resources and time to perform handovers. The proposed system was shown to result in fewer handovers, less interference, and less delay experienced by terrestrial users. This was achieved by setting appropriate coefficients for delay, interference, and handover in the reward function. In addition, concerning the energy efficiency of the moving UAV, a study proposed a system for mobile charging scheduling in distributed multi-drone networks [106]. They proposed a DL-based method to troubleshoot possible problems in distributed multi-drone networks effectively. The proposed system reduced the number of false bids made by drones by increasing the payment for those bids. That method resulted in a revenue-optimal auction, even without bid distribution among the drones. Table 4 provides a concise summary of the studies discussed in this subsection.

### 4.3. Autonomous Underwater Vehicles (AUVs)

Underwater communication is receiving a lot of attention from researchers lately. The increasing need for sensor applications and cellular communication through this environment encourages the importance of optimizing underwater communications. However, a crucial issue that needs to be considered is that the underwater environment can only occur by using optical or EM waves, which only occur in short-distance communication. Other than that, the water flow, movement of living things, uneven surfaces, and oceanic turbulence can cause a high level of multipath fading, reducing the quality of the transmitted signal [107]. An AUV is expected to serve the deployed nodes of the Internet of Underwater Things (IoUwT) by moving from one node to another to provide a better QoS, which resembles traditional mobile relaying [108]. AUV application can also be integrated with UAVs or IRS, where an AUV is well suited for carrying RIS to optimize the transmitted signals. Therefore, the AUV trajectory and limited energy are essential things to consider. Thus, further research is required to realize this strategy to support underwater wireless communication networks fully.

In [109], DRL has been proposed to find an AUV’s optimal trajectory tracking control. The proposed system has proven robust and effective in different kinds of trajectory tracking, while in [110], the authors proposed the asynchronous multithreading proximal policy optimization-based path planning (AMPPO-PP) and trajectory tracking (AMPPO-TT) algorithms for autonomous planning, tracking, and emergency obstacle avoidance in underwater vehicles. AMPPO-PP proved effective in planning paths around underwater communication by outperforming the classical path-planning algorithm and performing at the same level as the advanced sampling-based path-planning method. In contrast, AMPPO-TT is a trajectory-tracking algorithm that provides good tracking performance in three-dimensional coastline detection scenarios. Another study applied RL-based methods to control the underwater vehicle by redesigning the cost function, which allowed the vehicle to avoid obstacles smoothly [111]. The proposed system proved the effectiveness of completing the tracking task by avoiding obstacles. In comparison, the authors of [112] proposed an RNN with a convolution (CRNN) algorithm to overcome the obstacle avoidance issues. The CRNN solved the obstacle avoidance planning problem with fewer parameters and shorter computation times, leading to shorter paths and improved energy efficiency. Table 5 provides a concise summary of the studies discussed in this subsection.

### 4.4. Non-Orthogonal Multiple Access (NOMA)

The rapidly growing need for massive connectivity and the growth predictions of the use of emerging technologies on the 6G network makes spectrum efficiency a crucial issue that needs to be solved. NOMA is a promising and suitable technique to overcome that issue, due to its ability to provide highly efficient spectrum multiple access in a 6G wireless network [113]. In NOMA, several clusters are formed by a wireless terminal to transmit data over the same frequency channel. In addition, to prevent interference between clusters, each cluster implemented successive interference cancellation (SIC) [114].

In [115], unsupervised and supervised learning is implemented for spectrum sensing in NOMA communication. The proposed system achieved optimal power allocation between two primary users and accurate and effective spectrum sensing, while in [116], the authors focused on implementing LSTM-based DL models for signal detection. The results showed that the DL approach performed better than the SIC receiver, and was more robust than the limited radio resources. Other than that, ML can also be applied to NOMA communication to improve energy efficiency. In comparison, an energy-efficient ML power optimization algorithm was developed to meet QoS constraints in [117]. The proposed system significantly minimized energy consumption in a network while maintaining low complexity using an energy-efficient co-training-based semi-supervised learning (EE-CSL) algorithm. Due to its high spectral efficiency, the proposed system applied in the MIMO network achieved a more significant sum rate than conventional MIMO orthogonal multiple access, while in [118], the authors implemented a Double DQN (DDQL)-based RL to optimize the transmission power. The proposed DDQL algorithm reached the desired target value in 91% of the test cases. Compared to the sequential least squares programming algorithm (SLSQP) and trust-region constrained (TCONS) algorithms, the proposed DDQL algorithm significantly provided better results. Another study implemented a NOMA-based federated learning (DREAM-FL) system for client selection [119]. DREAM-FL proved to select more qualified clients with higher model accuracy than frequency division multiple access (FDMA)- and time division multiple access (TDMA)-based solutions, while in [120], the DL-based algorithm was implemented in the NOMA system for channel estimation. The LSTM-based DL algorithm is utilized to predict the channel coefficients. The bit-error-rate (BER), outage probability, sum rate, and individual capacity have verified that the proposed system provided reliable performance, even when cell capacity is increased. Table 6 provides a concise summary of the studies discussed in this subsection.

### 4.5. Millimeter-Wave and Terahertz Communications

By focusing on enhancing system performance, especially for throughput, 6G networks are expected to take advantage of the high-spread multi-band spectrum by allowing hundreds of gigabits per second to terabits per second links [121]. Other than that, for the sake of seamless connectivity of emerging technologies-based communication, higher spectrum frequency is something that can be considered to achieve fast and reliable communication, such as the combined use of mmWave band (30–300 GHz) and THz band (0.1–10 THz) [122]. However, these high-frequency communications will suffer from distance limitation, energy efficiency, physical layer improvement, and intense phase noise. The increasing frequency will result in higher spreading loss and stronger multipath fading losses. In addition, transceivers that can transmit at high power in the THz band are not yet available, which means that THz communication has lower transmit power than mmWave communication systems. Traditional transmission techniques are difficult to apply directly due to their inability to overcome intense phase noise caused by radio-frequency impairments in higher frequencies [123,124].

Several studies have proposed their schemes and algorithms to overcome some of the problems in mmWave wireless communication. In [125], ML is used for low-complexity beam selection in mmWave MIMO communication by implementing a random forest classification (RFC) algorithm. The proposed system achieved the maximum uplink sum rate, which is similar to the sub-optimization method and significantly better than the SVM-based method. Furthermore, it converged faster than SVM-based methods, and nearly reached the optimal performance. The RFC-based method could especially reduce the complexity of the system by 99.8% with massive users, while in [126], the authors proposed a supervised ML algorithm to improve the blind handover success rate in sub-6 GHz LTE and mmWave bands. The proposed system predicts the success or failure of the handover using previous calculations. The results showed that the proposed system improved the inter-radio access technology (inter-RAT) handover success rate and no longer kept the session in the optimal band for an extended time. Therefore, it likely has a high chance of supporting the self-organizing network regarding high availability, bandwidth, low latency, and reducing degraded service in a handover time. In [127], the authors applied three unsupervised learning algorithms to cluster secondary users without knowing the number of clusters and degrading the primary user’s performance. Three unsupervised ML algorithms, namely K-means, agglomerative hierarchical clustering, and density-based spatial clustering of applications with noise (DBSCAN), were used in the THz-NOMA network. Based on the sum data rates results, the agglomerative hierarchical clustering outperformed the other two algorithms as the number of secondary users increased. Table 7 provides a concise summary of the studies discussed in this subsection.

### 4.6. Free Space Optics (FSO)

Optical wireless communication (OWC) techniques can be an alternative to the RF spectrum, especially on 6G networks and in the future, due to their available bandwidth [128]. FSO communication is a type of communication that uses light to transmit data through free space rather than through wired cables. Therefore, FSO is more versatile and flexible than traditional wired communication. However, the signal will experience much interference, which can reduce the quality of the transmitted signal, such as multipath fading, atmosphere turbulence, and others. A concise summary of the studies discussed in this subsection is described in Table 8.

The authors of [129] focused on avoiding the effects of amplified spontaneous emission (ASE) noise, turbulence, and pointing errors by predicting the FSO channel for different transmission speeds using CNN and SVM. The results showed that CNN outperformed the SVM in most cases, and similar results for the rest. The CNN regressor could accurately predict channels with ASE noise regardless of the transmission speed. However, the turbulence and pointing error prediction was more accurate for low-speed than high-speed transmission, while in [130], the authors focused on atmospheric turbulence problems in the FSO-MIMO communication system. The dense CNN (DCNN) algorithm, which is DNN with a convolutional layer, was implemented in the proposed system’s transmitter, receiver, and transceiver sides. The results showed that the proposed DL-based methods performed better than ML-based methods in the case of optimum performance and lower complexity. In comparison, the DL-based detector with 16 modulation orders is two times faster, three times faster for 64 modulation orders, and 7.5 times faster for 256 modulation orders than the ML-based detector.

In addition, in [131], the authors worked on a cognitive FSO communication network that offers some tantalizing advantages. For example, it can overcome the system complexity caused by the heterogeneity of supported services, applications, devices, and transmission technologies, while guaranteeing a high data rate and bandwidth. They developed an unsupervised-learning-based method to identify the number of concurrently transmitting users sharing time. The system could also be used to allocate bandwidth, time, and space resources more efficiently. Based on the empirical model, the number of communicating users was considered accurate when validated from four users, considering the number of samples and receiver sampling rate. The result achieved over 92% accuracy in differentiating simultaneously transmitting users, even in conditions of moderate atmospheric turbulence. Another study applied a supervised-learning-based ML method to estimate the transmission quality for multi-user FSO communication links [132]. They compared the performance of SVM, RF, K-NN, and ANN to evaluate the proposed system. The results confirmed that SVM achieved the highest accuracy by 92%, followed by RF and K-NN with comparable results, and ANN at the lowest with 84.2%, while in [133], the combination of generative neural networks (GNN) and CNN considered the effects of turbulent light propagation, attenuation, and receiver noise detectors. Those factors could degrade the quality of the received state, increase cross-talk, and decrease the accuracy of symbol classification. The results showed that the proposed system efficiently received improved signals that had deteriorated from those problems. It also showed improvements in CNN classification accuracy while implementing GNN.

### 4.7. Visible Light Communication (VLC)

VLC is one of the other types of OWC communications, which uses light-emitting diodes (LEDs) to transmit signals to receivers [134]. VLC has many advantages, including rich spectrum resources between 400 and 800 THz, robustness against interference, high confidentiality, affordable implementation costs, and has become the best method to achieve high speed and long-distance signals in underwater wireless communications [135,136]. A concise summary of the studies discussed in this subsection is described in Table 9.

A study aimed to prevent eavesdropping in a MISO-VLC system by developing a secure and efficient way using the Deep RL algorithm [137]. The system proposed two ways to control beamforming, namely RL-based MISO VLC and DRL-based MISO VLC beamforming control schemes. Those two schemes were used to derive the optimal beamforming policy and to efficiently and effectively deal with the high-dimensional and continuous action and state spaces. The results showed that the proposed system greatly increases the secrecy rate, decreases BER, and outperforms the zero-forcing beamforming than other existing algorithms, while in [138], gated recurrent units (GRUs) with a CNN prediction algorithm were proposed to jointly optimize UAV deployment, user allocation, and energy efficiency of VLC-enabled UAV-based networks. The combined algorithms could model the long-term historical illumination distribution and predict the future illumination distribution, which could solve the non-convex optimization problem in low complexity. The proposed system showed a great result by reducing the total transmit power by up to 68.9%, by enabling UAVs to determine their deployment and user allocation. Another study proposed a model-driven DL-nonlinear post-equalizer scheme to cope with severe channel impairments of OFDM communication [139]. The authors showed how to estimate the channel and detect symbols that worked in a VLC system. The result showed that the overall channel impairment of intensity modulation and direct detection was effectively compensated, and the distorted symbols were efficiently demodulated to the bit stream. Furthermore, the VLC systems demonstrated that the proposed scheme is robust and generalizable, which can work effectively in various conditions.

The authors of [140] focused on the effect of low-frequency noise on the signal quality of LED-based VLC communication systems. The problem was overcome by mapping the LED-VLC system as an ANN-based AE structure and introducing an in-band channel model (IBCM) channel modeling strategy. High SNR training data was obtained for ANN-based IBCM. Furthermore, the embedded in-band autoencoder (IBAE) and IBCM were trained to combat the precise-estimated channel impairment, while avoiding performance degeneration due to the influence of the strong low-frequency noise. It achieved speeds of up to 0.325 Gbps faster than another scheme, indicating robustness to bias, amplitude, and bitrate changes. Another effect of distorted signals due to the nonlinearity of LEDs is the peak-to-average power ratio (PAPR). In [141], the LSTM autoencoder (LSTM-AE) dealt with variable input sequential data and predicted variable length output sequences in OFDM systems. The proposed model reduces the PAPR of the transmitted signal without increasing the BER.

### 4.8. Mobile Edge Computing (MEC)

The traditional cloud computing model has been widely adopted in the last decade. Computation offloading can extend the usability of mobile terminals. However, sending data to a central cloud is expensive and adds overhead delays, which can reduce the QoS of each user and can cause heavy losses for service providers [142]. Moreover, recently, the increasing growth of mobile terminals and the considerable transmission distance between the remote cloud and the user has increasingly driven this problem [143]. MEC is a technology that can reduce latency, improve energy efficiency, and provide more resources for mobile devices by performing computing tasks at the edge of the wireless network.

In [144], the base station (BS) was equipped with MEC to optimize spectrum and transmit power allocation. A multi-stack RL algorithm was proposed to help BS optimize its resource allocation for different tasks, including adjusting subcarriers, transmit power, and task allocation schemes. The proposed system enhanced learning efficiency and convergence speed by tracking past resource allocation schemes and user data at each BS. Therefore, it was more efficient than Q-learning, requiring 18% fewer iterations and resulting in 11% less maximal delay for users, while in [145], the ML algorithm was used for multiuser MEC systems in a cognitive eavesdropping environment. The authors proposed an FL framework to improve the efficiency of offloading tasks, allocating bandwidth, and distributing computational resources. The framework took latency and power consumption into account. The task offloading and resource allocation were formulated into a Markov decision process problem, while the state and action spaces were designed with DQN-based RL. FL framework distributed the DQN scheme to be run by each user to reduce the communication overhead and protect data privacy. The proposed method improved performance by reducing latency and energy consumption, while ensuring more bandwidth and computational resources for higher task-priority users. MEC is suitable for processing IoT computing-intensive tasks where the generated tasks can be offloaded to MEC. In addition, MEC is a promising technology to provide services for massive IoT devices. However, acquiring system information comprehensively and accurately had become a challenge in offloading multi-edge servers. In [146], the DRL-based energy efficient task offloading (DEETO) algorithm was proposed to enhance the energy efficiency and workload balance among the edge servers. The DEETO algorithm was found to be more energy-efficient and reduce edge server workload compared to the other RL algorithms mentioned in the paper.

Apart from supporting IoT communications, MEC can also be applied to the implementation of other emerging technologies. In [147], the DDPG-based RL algorithm was applied to optimize the physical-layer security of the IRS-assisted MEC network. The proposed system allocated the offloading ratio, bandwidth, and computational abilities to users. The results showed that the DDPG scheme found a more efficient way to offload tasks, resulting in a lower total cost than the all-local scheme. Furthermore, the system demonstrated the ability to work well in the MEC network under various conditions. Besides the IRS, UAV communication has also received widespread attention in MEC systems. In [148], the authors proposed a single-agent scheme based on Q-learning and a MA scheme based on Nash Q-learning (NQL) algorithms to maximize the secure offloading of multi-UAV-assisted MEC networks. The system solved the optimization problem while considering the limitations of the secure offloading transmission rate, computing latency, power consumption, and task types. The proposed system demonstrated that the MA scheme was better at optimizing the offloading and achieving greater system utility than the single-agent and random-offloading schemes. In contrast, the MA-TD3 (MATD3) approach was proposed in [149] to design a joint strategy of trajectory, task allocation, and power management. The result showed that the total system cost was significantly higher while applying the proposed approach than the other optimization method mentioned. Besides that, the proposed UAV-assisted edge cloud adapts to be flexible and adaptable to changing conditions, making it a promising technology for future wireless networks that are expected to become increasingly complex and dynamic. Table 10 provides a concise summary of the studies discussed in this subsection.

Based on the discussion in this section, Table 11 shows the application of ML algorithms for emerging technologies that could be implemented in 5G/6G communications to overcome several issues.

## 5. Potential Challenges for 6G Network Requirements

The ITU-R workshop on IMT addressed several usage scenarios and key capability indicators for 2030 and beyond. These indicators include in the KPIs for 6G communication networks, which need to be considered to design the future communication network, including high throughput (Gbps/Tbps), extended coverage, low latency, and high reliability. Furthermore, the network should have the capacity to support the interconnection of terrestrial and non-terrestrial technologies for both sensing and communication needs, while also being highly efficient in its operations.

### 5.1. Throughput Improvement

Throughput is a critical performance metric for wireless networks, and the demand for higher throughput is growing with each generation of technology. 6G networks are expected to deliver extremely high throughput, up to more than 1 Gbps [150]. One of the technologies that can enhance throughput in 6G networks is m-MIMO. However, m-MIMO requires a large number of antennas, which may not be feasible for handheld devices. A more practical solution is optimizing the beamformer, which is the antenna array used to transmit and receive signals.

ML can potentially enhance beamforming optimization by scanning wireless CSI, which is constantly changing and inherently imperfect. In particular, RL, a type of ML well-suited for unstable environments, can accurately optimize CSI. By utilizing the RL algorithm to select the optimal communication channel, we can achieve a throughput that approaches the theoretical maximum, even in the presence of imperfect CSI.

Another potential solution to the challenge of realizing a Tbps wireless network is to implement an optical spectrum. Optical spectrum, which works through free space, has the potential to achieve Tbps speeds due to its ability to overcome the limitations of coaxial cables. Despite the existence of certain limitations, such as the effects of atmospheric turbulence, ASE noise, and channel error, these can be minimized by applying an ML algorithm. This algorithm can learn the statistical properties of the optical spectrum and create a model to predict and mitigate these effects. Therefore, this would enable reliable and efficient data transmission at Tbps speeds over the optical spectrum.

### 5.2. Coverage Extension

The ever-increasing demand for wireless communication is inversely proportional to the decreasing availability of frequency capacity and the difficulty of constructing new BS. Implementing emerging technologies such as IRS and UAV can help address these issues by extending terrestrial BS coverage without overburdening the infrastructure. IRS, a software-defined meta-material that can be programmed to reflect wireless signals in desired ways, can extend the coverage of existing BS without requiring any new infrastructure, while UAVs are becoming increasingly sophisticated and can carry a variety of payloads, including BS equipment. This makes them a flexible and versatile tool for improving wireless coverage and a cost-effective way to provide in remote areas. Therefore, these technologies can be strategically positioned to transmit signals, such as providing a communication network in disaster-prone areas and on the high seas by implementing aerial BS or expanding the network in underwater and underground environments.

### 5.3. Ultra-High Reliability

The 6G cellular networks are expected to provide reliable and ultra-low latency communication. Especially with the presence of emerging technologies, the reliability of the 6G network is increasingly being anticipated. It is increased from 99.9 percent to 99.999 percent, which is ten times higher than 5G [151]. Despite the importance of ultra-low latency, it is difficult to achieve both low latency and high reliability. This is a significant challenge in developing architectures that support ultra-low latency, as well as spectral and energy efficiency [152].

AI-empowered technologies can be a solution to gain high reliability due to their predictable ability to solve complex problems and improve generative learning abilities [153]. AI-empowered technologies can be used to improve the reliability and availability of 6G networks in several ways. One of them is a single computing core that combines high-performance computing (HPC) and AI. HPC is the use of computers to solve complex problems that would be too time-consuming to solve using traditional methods. A combination of HPC and AI could help the system develop new algorithms to better manage traffic, optimize the use of network resources, and detect and prevent problems without human intervention [154,155]. It can predict and prevent network failures by analyzing historical data and identifying patterns that could lead to them. This information can then be used to take preventive measures, such as adjusting network settings or deploying additional resources. It can also be used to develop self-healing capabilities for 6G networks. Thus, these features could make the network more reliable by reducing the number of outages and the load on the network.

However, there are some challenges that need to be addressed in order to develop a single computing core. The development of new hardware that can combine the capabilities of HPC and AI is a major challenge, due to their need to be able to handle the high processing power and memory requirements of HPC. Furthermore, the software needs to be able to scale to large networks, handle a wide variety of data traffic, and protect the network from cyberattacks which can exploit the vulnerabilities of a single computing core.

In linear technologies with AI, which need to select information to avoid privacy concerns carefully, FL is one of the keys to this drawback, due to its ability to allow each user to run the learning algorithms individually without exchanging personal data. Other than that, as AI technologies, the ability to make decisions should not be questioned. Therefore, the role of DL will be indispensable for the smooth realization of this technology.

### 5.4. Low Latency Communication

Due to the massive connectivity in the 6G network, which is predicted to increase from 5G leading to unbearable latency, one of the demand requirements for the 6G network is URLLC, which requires latency of less than 0.1 ms [156]. There are two main challenges to achieving URLLC in 6G networks. First, the limited frequency bands available for cellular networks make it difficult to increase spectrum utilization rates [157]. Second, future communication networks may not be able to meet the latency, reliability, and other QoS requirements of URLLC, such as spectrum efficiency, energy efficiency, capacity, and network coverage [158].

ML algorithms offer a promising solution to these challenges. ML algorithms can be used to optimize network resources, such as spectrum and power, and to predict and prevent network failures, which would improve the network’s overall efficiency and reduce latency. In addition, ML algorithms can be used to develop self-healing capabilities, as mentioned in the last subsection. This is crucial for URLLC, as even a small amount of latency can significantly impact the applications’ performance. Even though the potential benefits of ML for URLLC are significant, and it is likely that ML will play a significant role in developing 6G networks, continuous research is needed on implementing ML to support the realization of optimal URLLC communication in various scenarios.

Other than that, as mentioned before, AI-empowered technologies can be implemented to improve reliability. They can also be used to achieve low-latency communication, such as predictive analytics to predict future traffic patterns. The predictive information will be used to adjust network settings and optimize routing, which would help reduce latency. Additionally, it can also reduce the latency by caching data in 6G networks, meaning frequently accessed data would be stored close to the user. Thus, applying the ML algorithm is one of the promising technologies to overcome these complex problems. Continuous research is needed on implementing ML to support the realization of optimal URLLC communication in various scenarios.

### 5.5. Energy Efficient Communication

The widespread adoption of emerging technologies in 6G communication networks is expected, which raises concerns about their impact on energy consumption. Even though realizing zero-energy IoT is very challenging, energy efficiency should be a critical issue in 6G networks. m-MIMO is expected to be a key technology in 6G networks, and emerging technologies such as IRS, UAVs, and AUVs are expected to play a significant role in m-MIMO communication. While MIMO can improve both spectral and energy efficiency [159], power consumption is a critical issue that must be considered when implementing emerging technologies-aided wireless communication. This is because emerging technologies are often more complex and require more power.

Likewise, the IRS mostly requires an energy supply due to the lack of power amplifiers [160]. UAVs and AUVs, on the other hand, consume energy when they move around and forward signals [161,162]. As a result, an energy-efficient mechanism must be considered to address this issue. This could involve developing a suitable optimization method to minimize power consumption or implementing wireless power transfer to recharge the required energy.

### 5.6. Interconnection of Terrestrial and Non-Terrestrial Technologies

Terrestrial and non-terrestrial technologies are being considered for use in 6G networks, because the interconnection of both technologies is one of the key enablers for a wide range of applications and benefits. However, some of the challenges need to be addressed in order to achieve seamless integration and to overcome some complex challenges, due to some issues such as heterogeneity, mobility, and security.

The upcoming 6G networks are predicted to be heterogeneous and support high mobility, comprising a range of disparate technologies, such as mmWave, THz, and m-MIMO. Network slicing, full duplex, and beamforming could also be implemented to address the challenges. Network slicing allows for the creation of virtual networks within a physical network, which will enhance spectrum efficiency and make it easier to interconnect terrestrial and non-terrestrial networks. By implementing these technologies, the interconnection of terrestrial and non-terrestrial technologies in 6G networks can be achieved, and it will allow for a wide range of new applications, such as VR, AR, self-driving cars, and critical infrastructure.

However, seamless integration will require new security protocols, as it will be necessary to ensure the data is secure as it moves between different networks. Other than that, standardization and cost should be the other issues that need to be considered. There is currently no agreed-upon standard for 6G networks, which makes it hard to confirm that different networks will be able to work together seamlessly. Furthermore, the cost of implementing seamless integration for 6G networks could be high, making it difficult for some businesses and organizations to adopt this technology.

### 5.7. Sensing and Communication

The 6G cellular networks are anticipated to utilize the broad spectrum of multiple frequency bands to enhance data transfer rates. This will be achieved through the new radio access technologies and the exploitation of the unique characteristics of the sub-THz spectrum. The sub-THz spectrum refers to the portion of the electromagnetic spectrum that lies between the microwave and infrared bands. This spectrum has a large amount of unused bandwidth, which can be used to achieve higher data rates [163,164].

In addition, the use of the sub-THz spectrum can lead to the development of integrated sensing and communication technology. This technology considers the entire communication system as a sensor and enables a wide range of new services, such as environmental reconstruction and high-accuracy imaging [165]. Localization obtained from sensing could enhance communication performance, such as improving beamforming, traffic routing, directing radio waves in a specific direction, reducing interference, and improving the SNR value [166,167].

Other than that, embedded space building (ESB) is another key technology that will be explored for 6G networks. ESB refers to the use of wireless networks to create virtual spaces that can be accessed by users. It involves embedding small, low-power sensors and actuators in the environment to create a distributed network of devices [168,169]. ESB can be used to create virtual space to support gaming, education, environment and healthcare monitoring, and training. It can provide users with a more immersive and realistic gaming experience, a more interactive and engaging learning experience, and provides trainees with a safe and realistic environment. It can also be used to monitor environmental changes and patient health, in order to provide remote tasks.

However, the sub-THz spectrum and ESB also pose some challenges, such as being easily absorbed by water vapor and oxygen, which limits their range. Furthermore, the use of integrated sensing and communication technology will require the development of new hardware and software. Other than that, there is no common standard for ESB yet, and the expected new services and applications, which implement high-definition virtual reality, augmented reality, and autonomous driving, are still being developed. Therefore, even though using the sub-THz spectrum and developing ESB offers beneficial things for communication networks, it still requires further research to fully implement it in a real-world scenario.

### 5.8. Secure Communication

Linearly with the high data traffic expected to occur in 6G communications, data security risks are also likely to increase due to wireless transmission characteristics [170]. Data privacy and security are crucial requirements for 6G communication. Besides protecting communication privacy for both parties, data security also affects the quality of the signal received by the receiver. Communication over the Internet is susceptible to cyber-attacks from malicious users, such as eavesdropping on important information being shared by others. In another scenario, jamming is a major security threat to wireless networks. It occurs when malicious parties intentionally transmit signals that interfere with legitimate communication or capture network bandwidth, thereby leaking vital information shared between communicating parties. Additionally, attackers may share or broadcast incorrect information to conduct data integrity attacks [171].

Therefore, we need security measures to those challenges while maintaining the security of wireless communication and protecting the data privacy and security of users who are communicating in the network. Strong authentication or encryption will be essential to prevent unauthorized access to 6G networks. This could be achieved using techniques such as biometric authentication or multi-factor authentication. Physical layer security (PLS)-based secret key generation is a promising new technique for securing communications and reducing shared information for eavesdroppers due to its more secure and efficient potential than traditional cryptographic schemes. Nevertheless, using PLS may be hindered by NLoS propagation when channel estimation is constrained by low SNR, high bit agreement rates, and low secret key rates (SKR) [172].

Network security monitoring is also needed to detect and respond to security threats, which can be achieved using techniques such as intrusion detection systems (IDS) and intrusion prevention systems (IPS). While specific to jamming problems, there are several ways that can be implemented to deal with jamming attacks. Frequency hopping is a technique where the network periodically changes its frequency [173]. It will make it difficult for attackers to keep up with the network and maintain the jamming signal. Implementing AI technology that can identify suspicious signals and dynamically adjust the modulation scheme used by networks could also help to ensure communication even in the presence of jamming. Spread spectrum and MIMO are also other techniques that can be implemented to improve the robustness of the network to interference [174,175]. Other than that, it is important to provide security education and raise awareness among all users, which will help users understand the potential security risks and acquire the fundamental skills to protect themselves.

In addition, quantum computing technology is an advanced method that is being developed to create secure communication. Compared with the classical binary-based communication systems that use bits either 0 or 1 to represent information, quantum communication uses qubits, which can be in a superposition of 0 and 1. This means that qubits can represent both 0 and 1 simultaneously, making it much more difficult to eavesdrop on [176,177]. It makes quantum communication carry a much more significant amount of information than classical communication systems [178]. Furthermore, it could significantly improve the transmission quality, as it is less susceptible to noise and interference, because qubits are not affected by the same physical processes as bits. Quantum communication also offers another potential benefit, which is absolute randomness and security. It is inherently secure because the act of eavesdropping will collapse the quantum state of qubits, which will be instantly detectable by the legitimate parties.

By implementing those measures, it could ensure a safer and more secure environment for all parties involved. Therefore, further research focusing on optimizing the parameters of each measure is required due to the limited research exploring this issue. Meanwhile, the development of quantum communication is still in its early stages, which also requires further research to implement it in real-world situations.

## 6. Conclusions

In the realm of communication networks, integrating emerging technologies is a critical factor to consider, especially in developing the forthcoming 6G network. These technologies can significantly boost the speed, security, and overall quality of communication services, even in locations or circumstances deemed challenging or hazardous to access. By leveraging cutting-edge technologies, 6G networks can offer unparalleled performance and reliability, making them a key enabler of a wide variety of applications and use cases in the future. However, implementing emerging technology-assisted wireless communications proved challenging for the model-driven approach, due to its inaccuracy. Furthermore, the high overhead and computational complexity of major emerging technologies hindered the system’s optimization, which conventional mathematical solutions cannot resolve. Therefore, ML algorithms, which are well-known and proven for their reliability in solving complex problems, are the leading solution for these concerns.

It is of utmost importance to create efficient algorithms and techniques that can cater to the needs of the upcoming 6G network. The 6G network is expected to have high requirements in terms of throughput, connectivity reliability, and energy efficiency. It is imperative to tackle these needs without the burden of high workloads and time complexities, as doing so is critical to the success of the network. Therefore, this article provides a comprehensive review of implementing ML, DL, RL, and DRL algorithms to optimize some of the difficulties that every emerging technology may face to meet the 6G network requirements. This study has revealed that the application of ML algorithms can effectively address a broad range of challenges related to spectral and energy efficiency, throughput, computational reduction, and the establishment of reliable and secure communication channels. However, despite their success in previous studies, it is essential to note that further research is necessary to fully harness the potential of these tools in advancing innovation. At the end of this study, we provide possible ML approaches to effectively address other challenges that may be presented in 6G network technology, such as extending network coverage, minimizing latency issues, connecting terrestrial and non-terrestrial technologies, and integrating the latest advances in sensing and communication technologies. Our recommendation aims to stimulate further discourse and exploration within this critical development area.

## Figures and Tables

**Table 1:** List of works surveyed on the implementation of ML for 6G communication networks.

References	Year	Limitations and Contributions
[28]	2021	ML algorithm for application and infrastructure layers in 6G network
[29]	2021	ML algorithm to meet ultra-low latency communication requirements
[30]	2022	ML algorithm-aided m-MIMO communication for 5G network
[31]	2022	DL algorithm for semantic communication in 6G network
[32]	2023	ML algorithm for integrated sensing and communication
[33]	2023	RL algorithm for RIS communication
Our work	2023	ML algorithms for emerging technologies to meet the 6G network requirements

**Table 2:** Summary of the ML techniques.

Category	Algorithms	Concept	Advantages	Limitations
Supervised learning	Linear Regression	Predicts continuous output based on input features	Easy to implement	Assumes a linear relationship between features and target
	Logistic Regression	Predicts binary or multi-class outcomes using logistic function	Easy to implement and interpretable results	Assumes linear decision boundaries
	Decision Trees	Creates a tree-like structure to make predictions	Intuitive and easy to interpret, faster computation, and capture non-linear relationships	Prone to overfitting
	Random Forest	Ensemble of decision trees to improve prediction accuracy	Reduces overfitting compared to individual trees, and effectively handling noisy and missing data	Computationally expensive during training and slower computation
	Gradient Boosting	Boosts weak learners (usually decision trees) sequentially	High prediction accuracy	Sensitive to noisy data and outliers
	Support Vector Machine	Finds the optimal hyperplane for binary/multi-class classification	Effective in high-dimensional spaces	Requires proper selection of kernel functions
Unsupervised learning	K-Means Clustering	grouping data into clusters based on similarities	Simple and easy to understand	Requires pre-determined number of clusters (K)
	Hierarchical Clustering	Creates a tree-like hierarchy of clusters based on data similarities	No need to specify the number of clusters beforehand	Sensitive to noise and outliers
	Principal Component Analysis	Reduces dimensionality while preserving variance	Efficient for large feature spaces	Information loss due to dimensionality reduction
DL	ANN	A set of interconnected artificial neurons that process input data	Suitable for complex tasks like image recognition	Prone to overfitting, especially with small datasets
	DNN	Fully connected NN with more than one hidden layer	Can learn complex features and patterns	Longer training time, especially for deep architectures
	CNN	Multi-layer NNs with convolution layer connected to the previous layer	Highly effective in image and video analysis	Requires significant computational resources for training
	RNN	Multi-layer NNs trained using back-propagation method	Can handle sequential data and suitable for time-series and NLP	Can suffer from vanishing gradient problems, computationally expensive to train, and difficult to parallelize the computation
RL		Trains agents to make decisions in an environment to maximize rewards	Useful in sequential decision-making tasks, suitable for super complex data, maximizes behavior, provides a decent minimization of performance standards	Not preferable for a simple problem, high sample complexity and training time, highly depend on the reward function quality, and difficult to debug and interpret

**Table 3:** Summary of the applications of ML for IRS-aided communications.

References	ML Model Architecture	Contributions	Remarks
[91]	Two full-layer DNNs	Optimization of phase matrix and beamforming vector	Reduced the pilot overhead and provided performance very close to communication with perfect CSI
[92]	CNN architecture	IRS phase shift optimization and overhead reduction	Converged to near-optimal data rates using less than 2% of receiver’s location
[93]	LPSNet	Spectral efficiency	Achieved almost the same performance as the alternating optimization method with less computational complexity
[94]	Three full-layer DNNs	Spectral and power efficiencies	Could configure real-time phase shift while improved rate performance in low SNRs and provided higher EE
[95]	DL-based framework	Spectral and power efficiencies	Provided the low complexity iterative algorithm with guaranteed convergence at a relatively optimal level
[96]	TD3 algorithm	Transmit power efficiency	Reduced the transmit power with lower computation delay
[97]	PDS and PER schemes	The learning convergence rate and efficiency	Enhanced the secrecy rate and the satisfied QoS probability
[98]	MA-DRL	Optimization of secrecy rate and throughput	Significantly improved the secrecy rate and throughput

**Table 4:** Summary of the applications of ML for UAV-aided communication.

References	ML Model Architecture	Contributions	Remarks
[100]	RL approach	UAV trajectory	Superiority in terms of average localization error by considering the fixed amount of UAV energy consumption, path length, flying time, and velocity
[101]	ESN algorithm	Placement optimization, trajectory acquisition, and power-control	Predicted the user’s movement at high accuracy and provided a high quality of maintaining the trajectory and power control
[102]	DQN-based algorithm	UAV path planning and obstacle avoidance	Reduction of 50% of computation time and 30% of the path length
[103]	DEO algorithm	Energy optimization	Achieved WMAPE in less than 2% under the effect of a communication delay of less than 1 s
[104]	MFTRPO algorithm	Optimal UAV trajectory	Effective in robustness and superiority in energy efficiency
[105]	ML-powered H-RRM scheme	Resource allocation and handover management	Outperformed the number of handovers, interference incurred, and delay experienced by setting coefficients for delay, interference, and handover
[106]	Two full layer DNNs	Energy efficiency of the moving UAV	Provided fewer false bids made by drones and revenue-optimal auction without bid distribution among the drones

**Table 5:** Summary of the applications of ML for AUV-aided communication.

References	ML Model Architecture	Contributions	Remarks
[109]	DRL algorithm	AUV optimal trajectory	Robust and effective in different kinds of trajectory tracking
[110]	AMPPO-PP and AMPPO-TT algorithms	Autonomous planning, tracking, and emergency obstacle avoidance	Outperformed the classical path-planning algorithm and advanced sampling-based path-planning algorithm
[111]	RL-based methods	Tracking and obstacle avoidance	Effective in completing the tracking task by avoiding obstacles
[112]	CRNN algorithm	Obstacle avoidance	Avoided obstacles with fewer parameters and shorter computation times, provided shorter paths, and improved energy efficiency

**Table 6:** Summary of the applications of ML for NOMA communications.

References	ML Model Architecture	Contributions	Remarks
[115]	Combined unsupervised and supervised learning	Spectrum sensing	Provided an accurate and effective spectrum sensing while maintaining optimal power allocation
[116]	LSTM-based DL models	Signal detection	Outperformed the (SIC) receiver and the limited radio resources
[117]	EE-CSL algorithm	Power optimization	Significantly minimized energy consumption for low computational complexity and achieved more significant sum rate than conventional MIMO orthogonal multiple access
[118]	DDQL-based RL algorithm	Transmission power optimization	Converged successfully in 91% of the test cases with a value better than the target, and performed better than SLSQP and TCONS algorithms
[119]	DREAM-FL scheme	Client selection	Provided more qualified clients with high model accuracy than FDMA- and TDMA-based solutions
[120]	LSTM-based DL algorithm	Channel coefficients prediction	Provided reliable performance even when cell capacity is increased

**Table 7:** Summary of the applications of ML for mmWave and THz communications.

References	ML Model Architecture	Contributions	Remarks
[125]	RFC algorithm	Low-complexity beam selection	Achieved better the maximum uplink sum rate, converged faster than the existing methods, and saved 99.8% of the complexity with for massive users
[126]	Supervised ML algorithm	Blind handover success rate prediction	Improved the inter-RAT handover success rate, kept the session in the optimal band, had a high chance of supporting the self-organizing network
[127]	Unsupervised ML-based user clustering algorithms	Secondary user clusterization and data rates improvement	The agglomerative hierarchical clustering outperformed the K-means and DBSCAN algorithms as the number of secondary users increased

**Table 8:** Summary of the applications of ML for FSO communications.

References	ML Model Architecture	Contributions	Remarks
[129]	CNN and SVM algorithms	Channel prediction	CNN outperformed the SVM, predicted channels with ASE noise well, and provided an accurate prediction for turbulence and pointing error in low-speed transmission
[130]	DCNN algorithm	Atmospheric turbulence problems detection	Achieved the optimum performance with low complexity, 2×, 3×, and 7.5× faster for 16, 64, and 256 modulation orders, respectively
[131]	Unsupervised-based technique	Estimated the number of concurrently transmitting users sharing time, bandwidth, and space resources	Achieved over 92% accuracy in differentiating simultaneously transmitting users, even in moderate atmospheric turbulence
[132]	Supervised learning-based ML method	Transmission quality estimation	SVM achieved the highest accuracy of 92%
[133]	Combined GNN and CNN schemes	Transmission quality estimation	Efficiently received improved signals that had deteriorated and showed better classification accuracy

**Table 9:** Summary of the applications of ML for VLC communications.

References	ML Model Architecture	Contributions	Remarks
[137]	Deep RL algorithm	Beamforming control	Significantly increased the secrecy rate, decreased the BER, and outperformed the zero-forcing and other existing algorithms
[138]	GRUs–CNN prediction algorithm	UAV deployment optimization, user allocation, and energy efficiency	Solved the non-convex optimization problem in low complexity and reduced total transmit power by up to 68.9%
[139]	Model-driven DL-nonlinear post-equalizer scheme	Channel estimation and symbol detection	Successfully proved the robustness and generalization ability, compensated for overall channel impairment, and demodulated distorted symbols to bit streams
[140]	ANN-based AE structure	Low-frequency noise effect prediction	Achieved speeds up to 0.325 Gbps faster than another scheme, and robustness to bias, amplitude, and bitrate changes
[141]	LSTM-AE scheme	Sequential data input handling and sequential data output prediction	Significantly reduced the PAPR while maintaining BER

**Table 10:** Summary of the applications of ML for MEC communications.

References	ML Model Architecture	Contributions	Remarks
[144]	Multi-stack RL algorithm	Subcarriers, transmit power, and task allocations	Reduced iterations by 18% and the maximal delay by 11% among users, compared to the Q-learning algorithm
[145]	FL framework with DQN-based RL algorithm	Offloading ratio, bandwidth, and computational ability optimization	Reduced the latency and energy consumption, ensured more bandwidth and computational capability for the higher task priority users
[146]	DEETO algorithm	Energy efficiency and workload balance maximization	Improved the energy efficiency and minimized the edge servers’ workload
[147]	DDPG-based RL algorithm	Physical-layer security optimization	Made a lower total cost decision and demonstrated the ability to work well under various conditions
[148]	MA scheme based on NQL algorithm	Multi-UAV secure offloading maximization	Outperformed the single-agent and random-offloading schemes in a better manner and achieved larger system utility
[149]	MATD3 scheme	Trajectory design, task allocation, and power management	Proven adaptable to EU mobility, changes in communication and computing resources, and dynamics of computing tasks

**Table 11:** ML-based algorithms for 5G/6G applications.

ML-Based Algorithm	Definition	5G/6G Applications	References
Supervised learning	ML algorithm that requires input and output pairs in advance to train the model	Spectral efficiency	[115]
		Energy efficient communication	[117]
		Computational reduction	[125]
		Throughput improvement	[125]
		Reliable communication	[126,131,132]
Unsupervised learning	ML algorithm that finds pattern data without labeled input and predefined output	Computational reduction	[93]
		Spectral efficiency	[93,115]
		Throughput improvement	[127]
ANN	Collection of neurons at each layer with inputs working in the feed-forward structure	Reliable communication	[140]
		Computational reduction	[140]
DNN	Fully connected structure connected with neurons in each layer	Reliable communication	[91,95,139]
		Spectral efficiency	[94,95]
		Energy efficient communication	[94,95,106]
		Throughput improvement	[94]
CNN	Structure with the same weight for all links with the convolution layer connected to the local path in the previous layer	Throughput improvement	[92]
		Spectral efficiency	[129]
		Computational reduction	[130]
		Reliable communication	[132,138]
		Energy efficient communication	[138]
RNN	Multi-layer feed-forward NNs Trained using the back- propagation method which considers input, weights, and memory for each output layer	Energy efficient communication	[103]
		Reliable communication	[112,116,120]
		Throughput improvement	[120,141]
RL	ML algorithms that allow machines to continuously learn from their experience data sets to automatically make the most accurate decisions	Energy efficient communication	[96,101,104,118,144,145,146,149]
		Reliable communication	[96,100,101,102,105,109,110,111,119] [144,148,149]
		Secure communication	[97,98,137,147]
		Throughput improvement	[98]
		Computational reduction	[144,145,146]
		Spectral efficiency	[145]

## Data Availability

Not applicable.
